# Diagnostic Performance of Extraprostatic Extension Grading System for Detection of Extraprostatic Extension in Prostate Cancer: A Diagnostic Systematic Review and Meta-Analysis

**DOI:** 10.3389/fonc.2021.792120

**Published:** 2022-01-25

**Authors:** Wei Li, Wenwen Shang, Feng Lu, Yuan Sun, Jun Tian, Yiman Wu, Anding Dong

**Affiliations:** ^1^ Department of Medical Imaging, Jiangsu Vocational College of Medicine, Yancheng, China; ^2^ Department of Radiology, Wuxi No. 2 People’s Hospital, Wuxi, China; ^3^ Department of Burn and Plastic Surgery, 71st Group Army Hospital of People’s Liberation Army of China, Xuzhou, China; ^4^ Department of Basic Medicine, Jiangsu Vocational College of Medicine, Yancheng, China

**Keywords:** prostate neoplasms, magnetic resonance imaging, diagnostic performance, extraprostatic extension, systematic review

## Abstract

**Purpose:**

To evaluate the diagnostic performance of the extraprostatic extension (EPE) grading system for detection of EPE in patients with prostate cancer (PCa).

**Materials and Methods:**

We performed a literature search of Web of Science, MEDLINE (Ovid and PubMed), Cochrane Library, EMBASE, and Google Scholar to identify eligible articles published before August 31, 2021, with no language restrictions applied. We included studies using the EPE grading system for the prediction of EPE, with histopathological results as the reference standard. The pooled sensitivity, specificity, positive likelihood ratio (LR+), negative likelihood ratio (LR−), and diagnostic odds ratio (DOR) were calculated with the bivariate model. Quality assessment of included studies was performed using the Quality Assessment of Diagnostic Accuracy Studies-2 tool.

**Results:**

A total of 4 studies with 1,294 patients were included in the current systematic review. The pooled sensitivity and specificity were 0.82 (95% CI 0.76–0.87) and 0.63 (95% CI 0.51–0.73), with the area under the hierarchical summary receiver operating characteristic (HSROC) curve of 0.82 (95% CI 0.79–0.85). The pooled LR+, LR−, and DOR were 2.20 (95% CI 1.70–2.86), 0.28 (95% CI 0.22–0.36), and 7.77 (95% CI 5.27–11.44), respectively. Quality assessment for included studies was high, and Deeks’s funnel plot indicated that the possibility of publication bias was low (*p* = 0.64).

**Conclusion:**

The EPE grading system demonstrated high sensitivity and moderate specificity, with a good inter-reader agreement. However, this scoring system needs more studies to be validated in clinical practice.

## Introduction

Prostate cancer (PCa) is the most common malignancy among males in Northern America and Europe, where one in nine men will be diagnosed with PCa at some point during their lifetime (1, 2). Compared with organ-confined disease (pT2), which can benefit from nerve-sparing surgical procedures, locally advanced disease [pT3, or extraprostatic extension (EPE)] is associated with a higher risk of biochemical recurrence and metastatic disease (3, 4). Despite that patients who underwent radical prostatectomy (RP) have shown high cancer-specific survival, they are suffering from postoperative erectile dysfunction and urinary incontinence (5). On the other hand, preservation of the neurovascular bundles (NVBs) can improve postoperative potency rates; however, increasing the risks of positive surgical margins then leads to biochemical recurrence and treatment failure (6). Thus, preoperative evaluation of EPE plays a crucial role in clinical management and treatment planning. Previously, varied clinical models and grading systems have been proposed for the prediction of EPE, including the Cancer of the Prostate Risk Assessment (CAPRA) score, Memorial Sloan Kettering Cancer Center (MSKCC) nomogram, and Partin tables (PT). Nonetheless, these risk stratification tools are lacking accuracy and are roughly correlated with final histopathologic results in clinical practice, with reported areas under the curve (AUCs) ranging from 0.61 to 0.81 (7–10).

In 2012, the European Society of Urogenital Radiology (ESUR) introduced Prostate Imaging Reporting and Data System (PI-RADS) for performing, interpreting, and reporting the PCa with multiparametric MRI (mpMRI) (11–13), which was widely applied in clinical practice (14–16). However, for localized advantage PCa of EPE, the ESUR PI-RADS demonstrated moderate diagnostic accuracy, mainly depending on radiologists’ own experience and short of reproducibility (17). Recently, a new scoring system termed the EPE grade has been proposed by Mehralivand et al. (18), the primary strength of which is simplicity and without needing to cooperate with complex imaging features. According to this grading system, grade 1 is defined as either curvilinear contact length ≥15 mm or capsular bulge and irregularity; grade 2 is defined as both curvilinear contact length ≥15 mm and capsular bulge and irregularity; and grade 3 is defined as visible EPE at MRI. Several studies showed that the EPE grading system has favorable diagnostic performance; however, this new guideline has not been evaluated systematically. Thus, in this study, we aimed to assess the diagnostic accuracy of using the EPE grading system for the prediction of EPE.

## Methods and Materials

This meta-analysis was in compliance with the Preferred Reporting Items for Systematic Reviews and Meta-Analyses (PRISMA) guidelines (19) and performed with a standardized review and data extraction protocol. A research question was established based on the Patient Index Test Comparator Outcome Study (PICOS) design criteria, as follows: what is the overall diagnostic performance of the EPE grading for prediction of EPE in patients with PCa? Our goal was to pool the sensitivity and specificity based on currently available retrospective and prospective cohort studies.

### Search Strategy and Selection Criteria

A computerized literature search of Web of Science, MEDLINE (Ovid and PubMed), Cochrane Library, EMBASE, and Google Scholar for studies applying the EPE grading system from December 2018 to September 2021, with no language restriction, was applied. The terms combined synonyms using for literature search, as follows: [(EPE) or (ECE) or (extracapsular extension) or (extraprostatic extension)] and [(PCa) or (prostate cancer) or (prostate carcinoma)]. Additional papers were identified from the most recent reviews and the reference lists of eligible papers.

### Inclusion Criteria

Studies would be included if they met the following eligibility criteria: 1) involved patients underwent MRI for assessment of suspected EPE, 2) with the EPE grading system for prediction of EPE in PCa, 3) reported sufficient information for the reconstruction of 2 × 2 tables to evaluate the diagnostic performance, and 4) with histopathological finding after RP as the reference standard.

### Exclusion Criteria

Studies would be excluded if any of the following criteria were satisfied: 1) studies with a too small sample of fewer than 20 participants, 2) studies using other guidelines or risk stratification tools rather than the EPE grading system, 3) not reported sufficient details for assessing the diagnostic performance, 4) studies with overlapping population, and 5) review articles, guidelines, consensus statements, letters, editorials, and conference abstracts. Two reviewers (WL and WS, with 8 and 5 years of experience, respectively, in performing systematic reviews and meta-analyses) independently evaluated all abstracts, subsequently reviewed full texts, and selected potential eligible articles; all disagreements were resolved through consensus in consultation with a third reviewer (AD).

### Data Extraction and Quality Assessment

The following information is extracted from each study: 1) demographic characteristics (sample size, patient age, prostate serum antigen (PSA) level, Gleason score or International Society of Urological Pathology (ISUP) classification, and number of patients diagnosed with EPE using histopathology; 2) study characteristics (first author, publication year, affiliation and location, period of patient recruitment duration, study design, cutoff threshold, other scoring systems used, number of readers and corresponding experience, and blinding; 3) technical characteristics (MRI sequences, magnetic field strength, and coil type); and 4) diagnostic accuracy information (number of true positive, false negative, false positive, and true negative findings classified with diagnostic criteria). Data extraction was performed by one investigator (WL) and confirmed by a second investigator (WS), with disagreements resolved by consensus after discussion with another one (AD). The methodologic quality of included studies was assessed with the Diagnostic Accuracy Studies-2 tool (20).

### Data Synthesis and Analysis

Heterogeneity among included studies was summarized with the inconsistency index (*I* ^2^) and *Q* test: for value between 0% and 40%, unimportant; between 30% and 60%, moderate; between 50% and 90%, substantial; and between 75% and 100%, considerable (21). Pooled sensitivity, specificity, positive likelihood ratio (LR+), negative likelihood ratio (LR−), diagnostic odds ratio (DOR), and their 95% CI were calculated with the bivariate model (22, 23) and then graphically presented in the forest plots; the area under the hierarchical summary receiver operating characteristic (HSROC) curve was calculated as well. In addition, we constructed an HSROC curve with a 95% confidence region and prediction region to demonstrate the results (22, 23). Publication bias was evaluated using Deeks’ funnel plot and determined with Deeks’ asymmetry test (24). All analyses were conducted using STATA 16.0, and statistical significance was set at a *p*-value <0.05.

## Results

### Literature Search and Data Extraction

**Figure 1:**
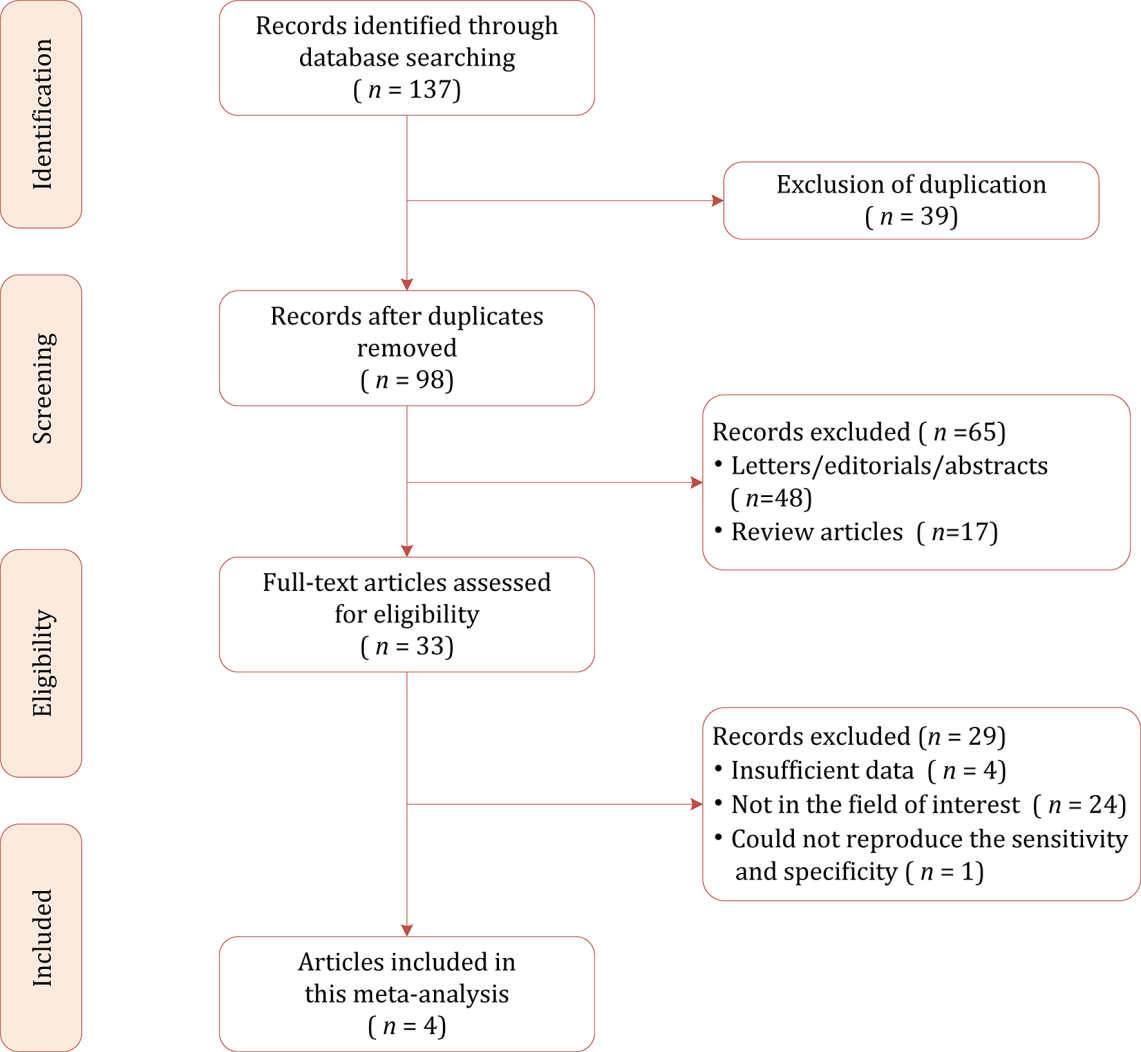
Study selection process for this systematic review and meta-analysis.

**Figure 1** shows the flowchart of the publication selection process. Our searches generated 137 relevant articles, of which 39 records were excluded for duplicates. After abstract inspection, 65 records were excluded, and a full-text examination was performed in the remaining 13 potentially eligible studies. A total of 29 studies were excluded due to insufficient data to reconstruct 2 × 2 tables, not in the field of interest, and could not reproduce the sensitivity and specificity. Consequently, a total of 4 studies comprising 1,294 participants were included in the present meta-analysis (18, 25–27).

### Characteristics of the Included Studies

The demographic characteristics are presented in **Table 1**. The sample size of the study population ranged from 130 to 553 patients, with a mean age of 60–65 years. Histopathological results after RP revealed that EPE was presented in 22.6%–48.5% of patients. The mean PSA levels of participants ranged from 6.28 to 9.95 ng/ml, with an ISUP category of 1–5. Concerning study design, only 1 was prospective, and all the remaining 3 were retrospective in nature. In all studies, MRI sequences of T2-weighted imaging (T2WI), dynamic contrast enhanced (DCE), and diffusion-weighted imaging (DWI) sequences were used. Regarding the cutoff, 1 study reported the outcomes of 3 thresholds (EPE grades ≥1, ≥2, and ≥3) (18), whereas the remaining studies only reported the outcome of a cutoff threshold ≥1. Aside from the EPE grading system, diagnostic accuracy of a quantitative assessment of the length of capsular contact (LCC) and in-house Likert scale were reported by 2 studies (18, 25, 26). In all studies, the MRI images were interpreted by 2 radiologists independently with experience of 2–15 years. The inter-reader agreement calculated with kappa values was reported by 3 studies, which ranged from 0.47 to 0.88 (25–27). In 1 study, the MRI was performed with a 1.5-T scanner (25), whereas all the remaining 3 studies used 3.0-T scanners. In 3 studies, the readers were blinded to final pathology results; however, 1 study reported that the readers were aware that patients had PCa (26). The study characteristics are summarized in **Table 2**, and the key points of the included studies are summarized in **Table 3**.

**Table 1:** Demographic characteristics of the included studies.

First author	Country	Year	Period	Patient number	Malignancy	Age (year, mean ± SD)	PSA (ng/ml, mean or median)	ISUP
Mehralivand	USA	2019	Jun. 2007/Mar. 2017	553	125	60 ± 8	6.28 (0.21–170)	1–5
Reisæter	Norway	2020	Jan. 2010/Dec. 2012	310	80	63.6 (60–67)*	8.8 (6–13)	1–5
Xu	China	2021	Jan. 2015/Jan. 2020	130	63	64.21 ± 8.10	9.95 (2.78–83.02)	1–5
Park	Korea	2020	Jul. 2016/Mar. 2017	301	129	65 ± 7	7.55 ± 5.62	1–5

NA, not available; PSA, prostate serum antigen; ISUP, International Society of Urological Pathology. *Median, interquartile range.

**Table 2:** Study characteristics of included studies.

First author	Study design	No. of readers	Experience (years)	Magnet field strength	*b* values (mm^2^/s)	Coil	Blinded	Other guidelines	*κ*	Cutoff threshold
Mehralivand	Prospective	2	9/15	3.0 T	1,500/2,000	ERC	Yes	LLC	NA	≥1/≥2/≥3
Reisæter	Retrospective	2	≥10	1.5 T	0/50/400/800/1,200	ERC	Yes	Likert	0.47	≥1
Xu	Retrospective	3	2/4/7	3.0 T	0–2,000	NA	Yes	CAPRA score MSKCC	0.88	≥1
Park	Retrospective	2	3/15	3.0 T	0/50/500/1,000	Surface	Yes*	Tumor size/LLC/ESUR score/Likert scale	0.71	≥1

ADC, apparent diffusion coefficient; CAPRA, Cancer of the Prostate Risk Assessment; ERC, endorectal coil; EUSR, the European Society of Urogenital Radiology; LCC, length of capsular contact; MSKCC, Memorial Sloan Kettering Cancer Center nomogram; NA, not available.

*Aware that all patients had prostate cancer.

**Table 3:** Key points of the included studies.

Study	Key points
Mehralivand	Proposed a standardized grading system for the detection of EPE at mpMRI, which provides a graded quantifiable risk assessment of EPE. It is based on only a few imaging features, making it easy to teach, and it should be relatively easy to implement.
Reisæter	Compared with Likert, the EPE grade showed a trend toward increased sensitivity at the cost of decreased specificity, and there was no significant difference in AUC for predicting EPE.
	The EPE grade showed moderate inter-reader agreement.
Xu	Comparing the EPE grade with the CAPRA score and MSKCCn, the results showed that the AUCs were comparable among these 3 models.
	Compared with using CAPRA score and MSKCCn alone, the combination of EPE grades significantly improved their diagnostic performance. Nevertheless, there was no statistically significant difference between the three combined models and EPE grade by itself (all *p* > 0.05).
	The EPE grade showed perfect inter-reader agreement between radiologists;
Park	Compared the EPE grade with Likert scale, ESUR, and length of capsular contact.
	The EPE grade showed substantial inter-reader agreement and good diagnostic performance, and association with histopathologic tumor extension.

EPE, extraprostatic extension; mpMRI, multiparametric MRI; AUC, area under the curve; CAPRA, Cancer of the Prostate Risk Assessment; MSKCCn, Memorial Sloan Kettering Cancer Center nomogram.

### Quality Assessment

**Figure 2:**
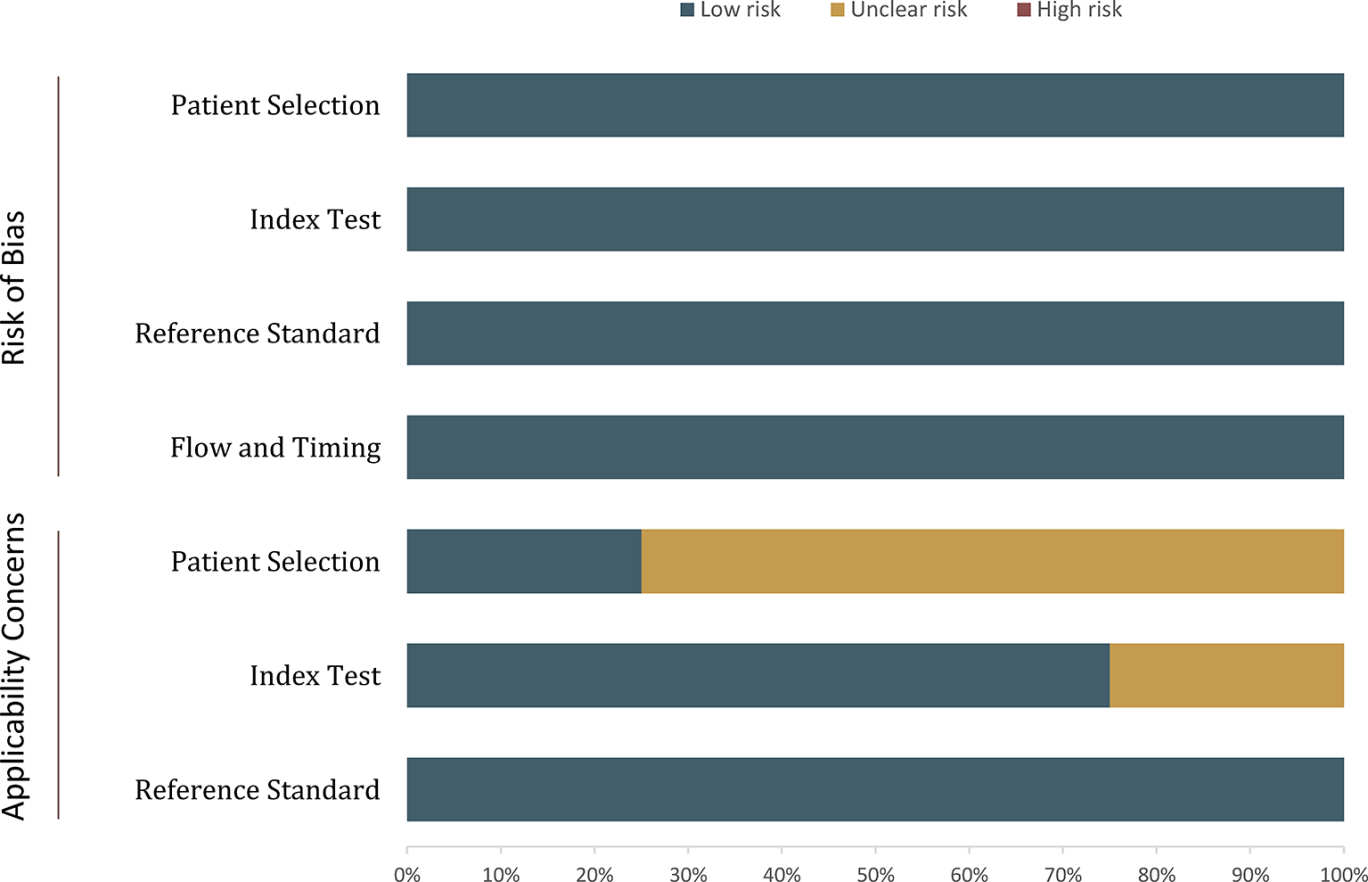
Grouped bar charts show the risk of bias and concerns for applicability of included studies.

Generally, quality assessment for included studies was high (**Figure 2**). However, concerning the patient selection domain, 3 of 4 studies were retrospective in study design (25–27). For the index test domain, one study reported that the radiologists were aware that patients were diagnosed with PCa and had undergone RP but were unaware of the final histopathologic finding (26). Concerning the two other domains, all studies were considered as low risk of bias.

### Diagnostic Accuracy of the Extraprostatic Extension Grading System

**Figure 3:**
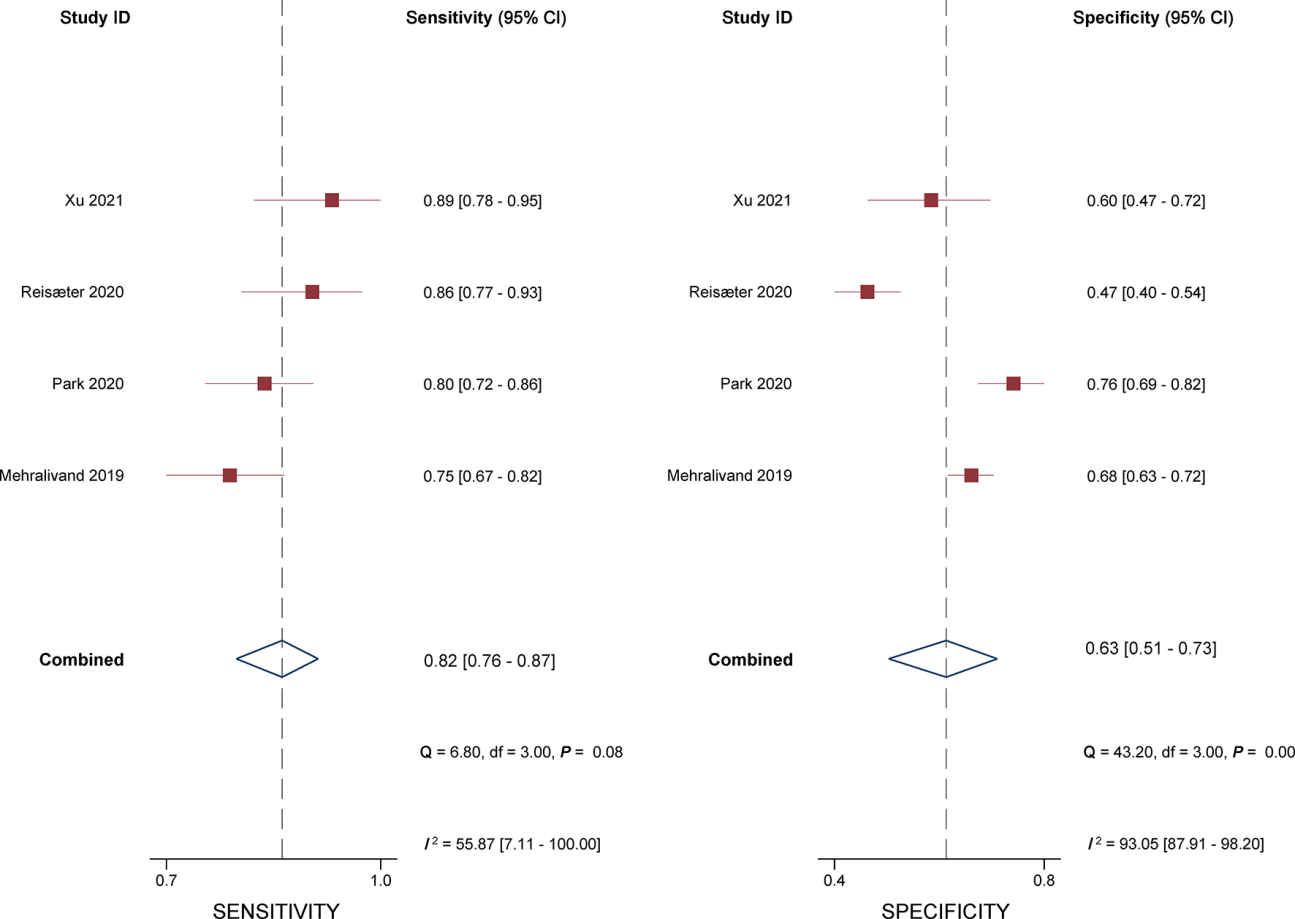
Coupled forest plot of pooled sensitivity and specificity.

**Figure 4:**
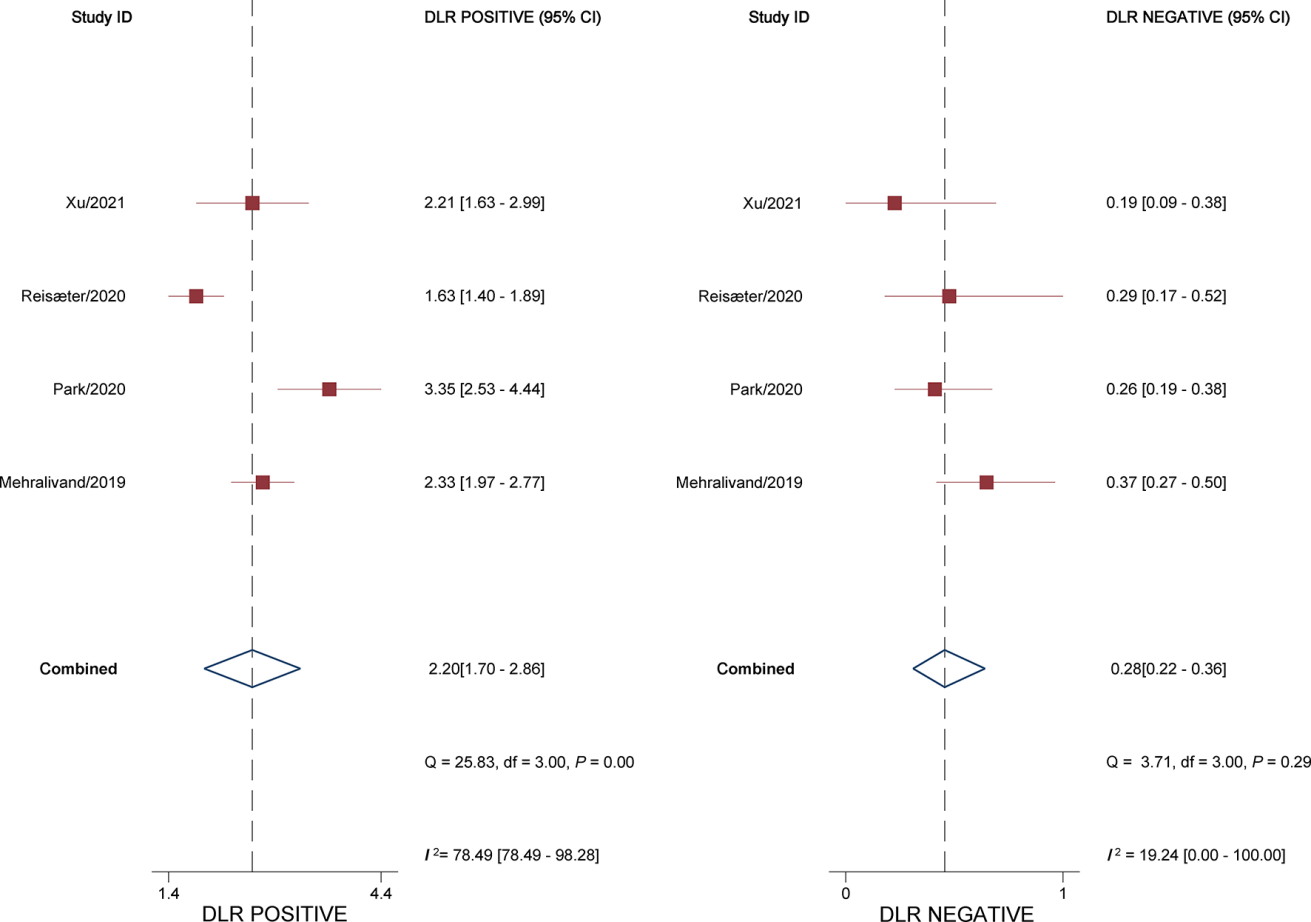
Coupled forest plot of pooled negative and positive likelihood ratios.

**Figure 5:**
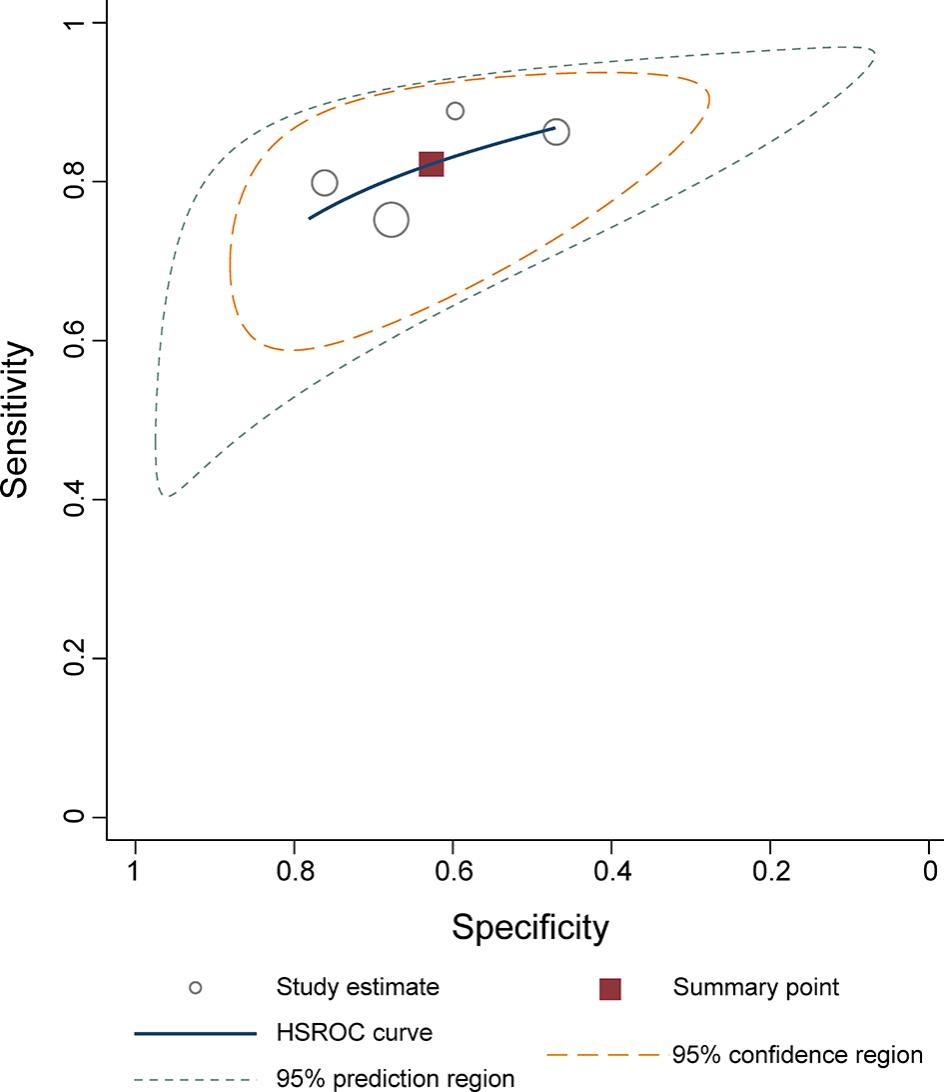
Hierarchical summary receiver operating characteristic plots with summary point and 95% confidence area for the overall.

**Figure 6:**
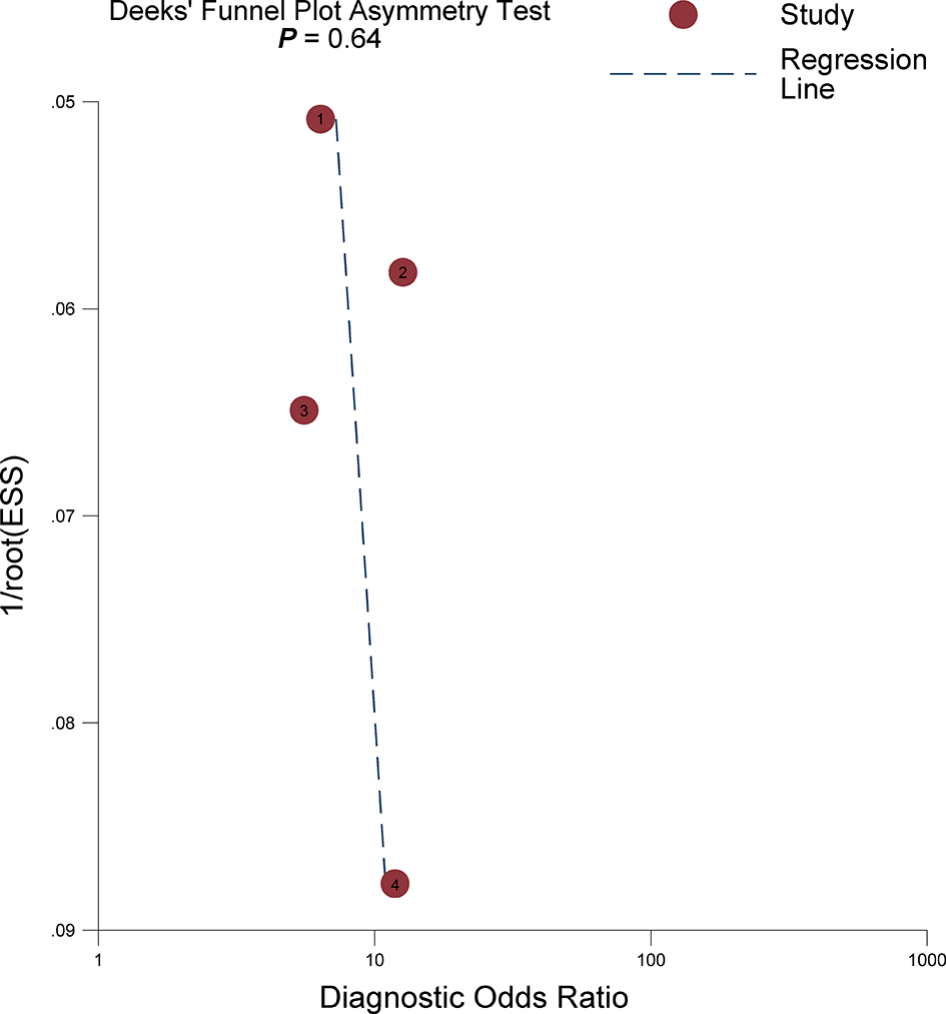
Deeks’s funnel plot. A *p*-value of 0.64 suggests that the likelihood of publication bias is low.

The sensitivity and specificity for individual studies were 0.75–0.89 and 0.47–0.76. Pooled sensitivity and specificity of 4 included studies combined were 0.82 (95% CI 0.76–0.87) and 0.63 (95% CI 0.51–0.73), respectively; the coupled forest plots are presented in **Figure 3**. Higgins’s *I* ^2^ statistics revealed moderate heterogeneity regarding sensitivity (*I* ^2^ = 55.87%) and considerable heterogeneity regarding specificity (*I* ^2^ = 93.05%). The pooled LR+ and LR− were 2.20 (95% CI 1.70–2.86) and 0.28 (95% CI 0.22–0.36), respectively, with a DOR of 7.77 (95% CI 5.27–11.44; **Figure 4**). The calculated area under the HSROC curve was 0.82 (95% CI 0.79–0.85). The large difference between the 95% confidence region and the 95% prediction region in the HSROC curve revealed heterogeneity between the studies, which is demonstrated in **Figure 5**. Deeks’ funnel plot and asymmetry test showed that there was no significant probability of publication bias among included studies, with a *p*-value of 0.64 (**Figure 6**).

### Discussion

In the current study, we assessed the diagnostic performance of the EPE grading system for predicting EPE in patients with PCa. Based on 4 studies, the pooled sensitivity and specificity were 0.82 (95% CI 0.76–0.87) and 0.63 (95% CI 0.51–0.73), with an area under HSROC of 0.82 (95% CI 0.79–0.85). Because of insufficient data, it is unfeasible to pool the summary estimates of inter-reader agreement; however, 3 studies reported that the *κ* values ranged from 0.47 to 0.88, indicating a moderate to substantial reproducibility among radiologists.

Previous conventional assessment of the 5-point EPE Likert scale (1 = highly unlikely, 2 = unlikely, 3 = equivocal or indeterminate, 4 = likely, and 5 = highly likely) have been employed widely in clinical practice, in which radiologists assign a score for the likelihood of EPE during MRI interpretation. However, the Likert scale primarily depends on radiologists’ personal patterns and experience and then lacks objective criteria, resulting in widely varied accuracy (28–30). A prior meta-analysis showed that the pooled sensitivity and specificity were 0.57 and 0.91 for detection of EPE with mpMRI (31); by contrast, the EPE grading system yielded higher sensitivity but lower specificity and with overall similar diagnostic performance. However, compared with previous MRI grading methods, the EPE grading system provided a standardized and simplified scoring system for the prediction of EPE, because it is based on only a few imaging features and is easy to teach and learn. Moreover, Xu et al. and Park et al. reported good inter-reader agreement while using the EPE grading system, and less experienced radiologists could benefit from this guideline and yield good diagnostic accuracy (26, 27). Nonetheless, the EPE grading system is still burdened with a subjective bias between radiologists due to some qualitative analyses (25).

For patients with EPE, aggressive surgery led to high cancer-specific survival but at the cost of a higher rate of urinary incontinence and erectile dysfunction, whereas preservation of the NVBs leads to a higher risk of positive surgical margin and biochemical recurrence, which then leads to treatment failure after RP. The optimal clinical decision is a trade-off, which needs accurate preoperative assessment of histopathologic EPE. An ideal scoring system should be based on precise definitions, is easy to apply in clinical practice, is robust, and has a high level of inter-reader agreement. For prediction of EPE, it should include both quantitative measures (apparent diffusion coefficient, tumor size, tumor volume, and LLC) and the qualitative criteria. The ESUR PI-RADS recommends reporting these features when evaluating mpMRI prostate examinations; however, it does not assign a likelihood of EPE based on a combination of these findings (11–13). Although the ESUR PI-RADS includes a discontinuous scale (1 = capsular abutment; 2 = not specified; 3 = capsular irregularity; 4 = NVB thickening, bulge, or loss of capsule; and 5 = measurable extracapsular disease) for prediction of EPE, only a few studies assessed its diagnostic performance. A recent meta-analysis showed that the pooled sensitivity and specificity were 0.71 and 0.76 (17). Nevertheless, the diagnostic results were extracted from more experienced readers or more accurate outcomes.

In recent years, quantitative metrics are intensively investigated for assisting the prediction of EPE, which includes LCC, ADC, tumor volume, and tumor size. These mpMRI quantitative metrics showed moderate-to-high diagnostic accuracy as an independent predictor for the detection of EPE. Nonetheless, various measurement approaches and tools, along with MRI techniques and sequences, result in widely varied optimal cutoff thresholds (32, 33). The EPE grading system recommends the quantitative metric of 15-mm curvilinear contact length as a threshold for evaluation of EPE; however, it was unclear how such threshold was derived. According to current evidence, the reported optimal threshold varied from 6 to 20 mm, with sensitivity of 0.59–0.91 and specificity of 0.44–0.88 (34). The lower cutoff value for predicting EPE will lead to higher sensitivity but at the cost of decreased specificity, and vice versa. In PI-RADS v2, a tumor size of 15 mm was recommended as the cutoff for the prediction of EPE, while some studies demonstrated that the optimal threshold was 16–18 mm (35, 36). Nevertheless, this quantitative assessment was not included in the EPE grading system.

There are some limitations to our study. First, the majority of studies included were retrospective in study design, leading to high risk regarding patient selection domain. Nevertheless, it is unfeasible to pool the summary estimates from prospective studies. Second, substantial heterogeneity was found across included studies, which affected the general applicability of our meta-analysis. However, it is impossible to perform meta-regression and subgroup analyses to investigate the source because there are merely 4 studies in total. Nevertheless, we applied a solid and robust methodology for this meta-analysis using the guidelines published by the Cochrane Collaboration. Third, our analysis was based on only 4 studies; therefore, the results should be regarded with caution, and large prospective studies are needed to validate this guideline in the future. In addition, because of insufficient information, we cannot perform direct comparisons between the EPE grades with other scoring systems.

## Conclusions

The EPE grading system demonstrated high sensitivity and moderate specificity, with a good inter-reader agreement. However, this scoring system needs more large prospective studies to be validated in clinical practice.

## Data Availability Statement

The original contributions presented in the study are included in the article/supplementary material. Further inquiries can be directed to the corresponding authors.

## Author Contributions

Guarantor of the article: JT. Conception and design: WL and WWS. Collection and assembly of data: FL and YS. Data analysis and interpretation: YMW, JT, and ADD. All authors contributed to the article and approved the submitted version.

## Funding

This study was supported by the Natural Science Foundation of Jiangsu Vocational College of Medicine (No. 20204112).

## Conflict of Interest

The authors declare that the research was conducted in the absence of any commercial or financial relationships that could be construed as a potential conflict of interest.

## Publisher’s Note

All claims expressed in this article are solely those of the authors and do not necessarily represent those of their affiliated organizations, or those of the publisher, the editors and the reviewers. Any product that may be evaluated in this article, or claim that may be made by its manufacturer, is not guaranteed or endorsed by the publisher.
